# Supplementary material for: Clinical Outcomes and Quantitative HBV Surface Antigen Levels in Diverse Chronic Hepatitis B Patients in Canada: A Retrospective Real-World Study of CHB in Canada (REVEAL-CANADA)
Source: Viruses. 2022 Nov 29;14(12):2668. doi: 10.3390/v14122668 (PMC9781785; doi:10.3390/v14122668)
Supplement: Supplementary file 1 [file viruses-14-02668-s001.zip › viruses-2000868-supplementary.pdf]

Supplementary Table S1: List of countries with ≥5% prevalence of HBV were considered endemic.[36]

Region	Countries with ≥ 5% prevalence of HBV.
WHO African Region	Angola, Benin, Burkina Faso, Burundi, Cameroon, Cape Verde, Central African Republic, Congo, Côte d'Ivorie, DR Congo, Equatorial Guinea, Ethiopia, Gabon, Gambia, Ghana, Guinea, Kenya, Liberia, Malawi, Mali, Mauritania, Mozambique, Namibia, Niger, Nigeria, Rwanda, Senegal, Sierra Leone, South Africa, South Sudan, Swaziland, Togo, Uganda, Tanzania, Zambia, Zimbabwe
WHO Region of the Americas	Haiti
WHO Eastern Mediterranean Region	Djibouti, Oman, Somalia, Sudan, Tunisia, Yemen
WHO European Region	Albania, Kazakhstan, Kyrgyzstan, Moldova, Romania, Tajikistan, Uzbekistan
WHO South East Asian Region	Bhutan, Thailand
WHO Western Pacific Region	China, Kiribati, Laos, Marshall Islands, Mongolia, Nauru, Niue, Papua New Guinea, Samoa, Solomon Islands, Tonga, Tuvalu, Vanuatu, Vietnam

Supplementary Table S2: Comparison of hepatic complications in individuals with HBsAg loss (N=237) compared to those who still have detectable HBsAg (n= 607). Continuous data are shown as mean (95% CI, n known). Categorical data are shown as mean % (n/n known).

	HBsAg loss (n= 237)	Detectable HBsAg (n= 607)	P value
Hepatic complications			
Cirrhosis	5.7% (13/230)	10.9% (61/558)	0.021*
Hepatocellular carcinoma	0.9% (2/231)	6.2% (34/549)	0.001*
Liver fibrosis			
TE kPa	5.9 kPa (5.3-6.5, 124)	6.0 kPa (6.0-7.2, 398)	0.277
>F2 fibrosis (TE >7.3 kPa)	13.7% (17/124)	18.3% (73/398)	0.233
>F3 fibrosis (TE >10.7 kPa)	8.1% (10/124)	7.3% (29/398)	0.774

[#] T-test was used for continuous data, chi-square tests were used for categorical data. P < 0.05 considered significant. For both continuous and categorical variables missing data are excluded.

Supplementary Table S3. Multivariable Analysis of Risk Factors for

variable	estimate	95% CI lower	95% CI upper	P value
(Intercept)	0.0055	0.000	0.050	0
Age >60 years	1.7093	0.619	4.718	0.300
Male	0.9946	0.356	2.777	0.991
Alcohol history	0.701	0.173	2.838	0.618
Smoking history	1.869	0.607	5.747	0.275
High risk activities	11.28	1.5734	80.862	0.0159*
Elevated ALT (>ULN)	0.918	0.347	2.422	0.863
HBeAg positive	1.0232	0.287	3.641	0.971
HBV DNA Detected	0.4787	0.156	1.466	0.197
> 2 Comorbidities	0.723	0.210	2.485	0.606
NAFLD diagnosis	1.085	0.335	3.516	0.891
Cirrhosis	5.6229	2.042	15.476	0.0001*
No treatment	8.232	1.005	67.393	0.0493*

Supplementary Table 4: Multivariate Analysis of Risk Factors for Cirrhosis

variable	estimate	95% CI lower	95% CI upper	p value
(Intercept)	0.01	0.00	0.38	0.01
Age >60 year	2.07	0.69	6.18	0.18
Sex Male	0.7044	0.232	2.132	0.535
Alcohol history	0.52	0.11	2.26	0.38
Smoking history	2.7985	0.790	9.907	0.110
Country of birth - Endemic	0.15	0.02	1.04	0.05*
Elevated ALT (>ULN)	1.60	0.56	4.53	0.37
HBeAg Positive	2.3714	0.665	8.445	0.182
HBV DNA Detected	0.313	0.084	1.155	0.0813
> 2 Comorbidities	1.75	0.54	5.65	0.34
NAFLD diagnosis	2.045	0.703	5.946	0.188
No treatment	16.4595	1.88	143.39	0.0112*

Supplementary Table 5. Linear Model of quantitative HBsAg in association with virological and clinical outcomes

variable	estimate	95% CI lower	95% CI upper	P value
(Intercept)	4.2314	1.708	6.754	0.001
> 60 years	-1.467	-2.270	-0.664	0.0004
Male	-0.1783	-0.8425	0.485	0.599
Country of birth Endemic	-0.6783	-2.229	0.873	0.392
Asian	0.1789	-1.721	2.079	0.853
Black	2.233	0.125	4.342	0.0387
Other	0.1445	-2.479	2.768	0.914
Alcohol	0.7304	-0.107	1.568	0.0887
Smoking	-0.513	-1.404	0.378	0.260
High risk activities	0.0469	-3.233	3.327	0.977
HBeAg Positive	1.48	0.5993	2.360	0.0011
Elevated ALT (ULN)	0.7193	0.0549	1.383	0.0346
HBV DNA Detected	1.6392	0.843	2.435	0.00007
> 2 Comorbidities	-0.1332	-1.076	0.810	0.782
NAFLD	-0.252	-1.047	0.543	0.535
HBV Treatment	1.6221	0.750	2.493	0.00031
Cirrhosis / HCC	-0.6976	-1.781	0.386	0.208
